# The Black *Aspergillus* Species of Maize and Peanuts and Their Potential for Mycotoxin Production

**DOI:** 10.3390/toxins2040399

**Published:** 2010-03-24

**Authors:** Edwin R. Palencia, Dorothy M. Hinton, Charles W. Bacon

**Affiliations:** 1 Department of Plant Pathology, University of Georgia, Athens, GA, USA; 2 Toxicology &amp; Mycotoxin Research Unit, USDA, ARS, Russell Research Center, P.O. Box 5677, Athens, GA, USA

**Keywords:** *Aspergillus niger*, *Aspergillus* section *Nigri*, black aspergilli, fumonisins, ochratoxins, mycotoxins

## Abstract

The black spored fungi of the subgenera *Circumdata*, the section *Nigri (=Aspergillus niger* group) is reviewed relative to their production of mycotoxins and their effects on plants as pathogens. Molecular methods have revealed more than 18 cryptic species, of which several have been characterized as potential mycotoxin producers. Others are defined as benign relative to their ability to produce mycotoxins. However, these characterizations are based on *in vitro* culture and toxins production. Several can produce the ochratoxins that are toxic to livestock, poultry, and humans. The black aspergilli produce rots of grapes, maize, and numerous other fruits and grain and they are generally viewed as post-harvest pathogens. Data are review to suggest that black aspergilli, as so many others, are symptomless endophytes. These fungi and their mycotoxins contaminate several major grains, foodstuffs, and products made from them such as wine, and coffee. Evidence is presented that the black aspergilli are producers of other classes of mycotoxins such as the fumonisins, which are known carcinogenic and known prior investigations as being produced by the *Fusarium* species. Three species are identified in U.S. maize and peanuts as symptomless endophytes, which suggests the potential for concern as pathogens and as food safety hazards.

## 1. Introduction

Fungi of the genus *Aspergillus* have a long history of associations with humankind. The genus was first described by Micheli in 1729 [1] to include those fungi with long stalks and spore heads that radiated in long chains from a central structure resembling an aspergillum, the brush-like structure used in religious ceremonies for sprinkling holy water. Species of this genus are common, originally applied to the first species of this genus, *A. glaucus* by Link [2]. They are extremely diverse in their habitats, and extraordinarily versatile in terms of their ability to produce secondary metabolites. Several are important human and plant pathogens, while others are useful in the production of several fermented food products highly regarded by humankind. Due to their metabolic versatility, species of this genus have great biotechnological potential and several are used for the production of numerous food and nonfood by-products. The plant pathogens are of concern not only for their ability to destroy several agronomically important food crops, but also due to their ability to produce several mycotoxins. These mycotoxins are associated with specific species or subgenera of the *Aspergillus* and are in general toxic to livestock, poultry, fish, and humans. 

Early attention was devoted to species of *Aspergillus* as causes of animal toxicities, mainly poultry [3]. Immediately after this report, it was established that this toxicity, described as the Turkey-X disease of peanuts, was caused by *A. flavus* and *A. parasiticus* [4]. Subsequent studies resulted in identifying the aflatoxins as the toxicological agent [5], which initiated mycotoxicology as a serious and complex problem of food safety. Continued studies resulted in distinguishing another class of mycotoxins produced by species of aspergilli, the ochratoxins, which along with the aflatoxins, were established as important carcinogenic mycotoxins. Ochratoxin A was discovered in maize by Van der Merwe *et al.* [6] as the mycotoxin produced by strains of *Aspergillus ochraceus* Whilh, and *Penicillium verrucosum*. One of the first reports for the natural occurrences of ochratoxicosis was in poultry, which consisted of five independent episodes including about 970,000 turkeys, two episodes of about 70,000 laying hens, and two episodes in about 12,000,000 broilers [7]. These observations served to establish ochratoxins as important toxins in agriculture, and in food safety. Recently the ochratoxins have been reported from several other species of *Aspergillus* sections *Circumdati* (*A. ochraceus* group), and by *Eurotium herbariorum,* a member of the *Aspergillus* section (*A. glaucus* group). Recently, the black species of *Aspergillus* have been shown able to produce the ochratoxins [8], which extends the geographic area of concern from the temperate zone to the tropical and sub-temperate zone due to the distribution of these black species.

The black aspergilli are commonly found as soil organisms decomposing dead plant residues [9] and they are pathogenic on several crops. As discussed below, the majority of the black *Aspergillus* species are associated with grapes, onions, maize, and peanuts, where they are cited as pathogens causing such diseases as peanut and maize seedling blight, and maize kernel rot. However, there are numerous examples of fungi associated with plants as symptomless endophytes, and there is evidence that this life habit may be practiced by the *Aspergillus* species as well. In association with several hosts, these symptomless endophytes have the capacity to either develop as pathogens or as saprophytes, and in either state become producers of mycotoxins. Symptomless expressions of several black aspergilli are indicated in the literature but nothing is indicated about their ability to produce mycotoxins and any associated pathology. Further, the black species of *Aspergillus* associated with any plant pathological problem was indicated in early publications as *A. niger,* the black species. In this review, we refer to *A. niger sensu strictu*, *i.e.*, *A. niger* var. *niger*, to designate or distinguish the present day discription from the older published accounts, which in most instances were applied *sensu lato* and will be referred to here as simply *A. niger*. While several cryptic species within this subgenus have been delineated, this recent taxonomic revision creates a large gap of knowledge of black *Aspergillus* species that are presently defined, and which is important to food safety and plant pathology. This review focuses on identifying the species of the subgenera *Circumdati*, the section *Nigri* (=*A. niger* group) (Table 1) encountered on cereals and other plants, with an emphasis on maize and peanuts in particular. The nature of the association with their hosts and their potential to produce the ochratoxins and other toxins relative to specific species are also reviewed. 

## 2. The Black *Aspergillus* Species

There are well over 190 number of *Aspergillus* species, and these can be conveniently separated into several distinct morphospecies, and several of these are based on colors according to the earlier classification of Raper and Fennell [10]. However, phylogenetic analyses of sequence data resulted in separating the *Aspergillus* genus into eight subgenera [11]. Following these analyses, the economically important species that produce the ochratoxins were divided to include those species of the subgenera *Circumdati*, the sections *Circumdati* (=*Aspergillus ochraceus* group) and *Nigri* (*A. niger* group). There are no known teleomorphic species of group *Nigri*. In recent years, members of the *Aspergillus* section *Nigri* have undergone an extensive taxonomic revision resulting in several new taxa, such as *A. niger* var. *niger*, *A. melleus*, *A. sulphureus*, *A. brasilensis*, *A. ostianus*, *A. petrakii*, *A. scletotium*, *A. carbonarius*, *A. aculeatus*, *A*. *japonicus*, *A. tubingensis*, *A*. *ibericus* and *Eurotium herbariorum* [12,13,14] (Table 1).

However, these new taxa have not been identified as to the responsible species in diseases of food crops, such as maize seedling blight, maize ear rot and seedling blight of peanuts. Further, any role they may play in the pathogenic expression of maize kernels and plants prior infected with *Fusarium verticillioides*, a common maize pathogen usually co-associated with black aspergilli as a symptomless infection, is unknown.

Klich [9] classic paper on the biography of the *Aspergillus* genus indicated that most of the species occurred in the tropical latitudes below 25 degree north and south, with greater than expected frequencies in the subtropical to warm temperate zones at latitudes between 26 and 35 degrees. This study suggested that species abundance peaked in the subtropics. This distribution is attributed to several biotic and abiotic interacting factors with the major factor temperature [9]. In general, the black species of aspergilli were found to occur more frequently in forest and cultivated soils and less frequency in desert soils. However, *A. niger* var. *niger* was uniformly distributed throughout the entire samples areas including forest, grassland, wetlands, deserts, and cultivated soils [9]. Thus, this documents this species as the most common species in both subgenera of *Circumdati.*

**Table 1:** Selected toxins isolated from black *Aspergillus* species, modified from Nielsen *et al.* [36].

Species^a^	Host^b^	Ochratoxin	Biologically active metabolites
*A. niger var. niger^c^*	Maize, peanuts, grapes, and grape products, coffee, tea, beans, spices^d^	+^i^	Fumonisin B2
			Fumonisin B4
			Nigragillin^e^, Malformin^f^
*A. carbonarius*	Grapes, java coffee bean	+	Carbonarones^g^
*A. tubingensis*	Arabica coffee beans	+	Malformin, nigranillin
*A. brasiliensis*	Grapes	-	Malformin
*A. acidus*	Raisins	-	Uk
*A. ibericus*	Grapes	-	Uk
*A homomorphus:*	Soil, nh	-	Secalonic acid^h^
*A. ellipticus*	Soil, nh	-	Terphenyllin^f^
*A. aculeatinus*	Arabica coffee bean	-	Uk
*A. aculeatus*	Green Coffee bean	-	Secalonic acid, Aspergillusol A^g^
*A. japonicus*	Grapes, maize, peanut	+	Cycloclavin
*A. uvarum*	Healthy grapes	-	Secalonic acid
*A. piperis*	Black pepper	-	Aflavinines^e^
*A. sclerotiicarbonarius*	Robusta coffee bean	-	Uk
*A. sclerotioniger*	Coffe bean		Aflavinines
*A. heteromorphus*	Soil, nh	-	Uk

^a ^*A. niger* var. *niger* consists of several synonym including *A. awamori, A. phoenicis, A. kawachii, A. saitoi, A. usamii, A. foetidus, A. citricus,* and *A. ficuum,* and oftentimes these synonyms are listed by several authors as varieties of *A. niger, i.e.,* *A. niger* var. *awamori,* and as such they are ochratoxin *A* producer [36]. ^b^Indicates the principle plant host associated with the species as a parasite if known, but does not preclude nor exclude soil and other saprophytic habitats, which is indicated as nh, no host known. ^c^Data indicated for this species may or may not reflect information on *A. niger sensu stricto*, rather the generalized descriptive placement of black aspergilli in this species complex. ^d ^The spices include isolations from various plant parts of black cumin, fennel, lime tree, absinthium, ginger, cinnamon, peppermint, carob tree, chamomile, saffron, curcuma, wormwood, rose, and lesser galangel [72]. ^e^Insectidal. ^f^Phytotoxic. ^g^Antibiotic. ^h^Weak mycotoxic activity. ^i^Symbols, +, -, present or absent; uk, unknown.

**Figure 1:**
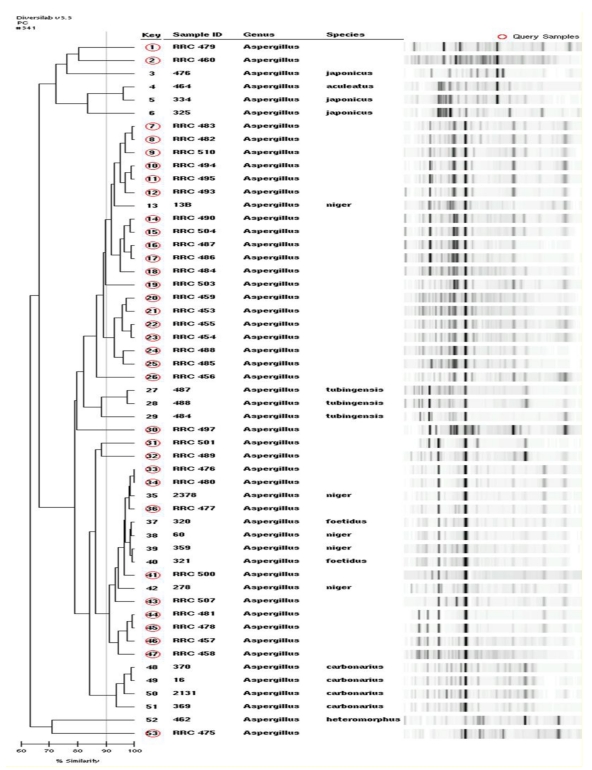
Dendrogram and gel-like images illustrating rep-PCR barcodes of 54 *Aspergillus* section *Nigri* isolates, designated RRC from corn and peanuts samples, analyzed along with reference species of black aspergilli by a rep-PCR barcoding procedure [15]. Queried sample numbers are indicated by circles under the key column of this figure and the identifications of each sample ID number is indicated in Table 2 [15].

In a recent survey of maize and peanut using rep-PCR to distinguish morphotypic and molecularly derived species (Figure 1), several basic black *Aspergilli* were distinguished in peanuts and maize [15]. The survey was designed to analyze for endophytic species of these two plants using surface disinfection of kernels and plant parts. This survey indicated that several species were present in these two plants as seedborne systemic and endophytic infections (Table 2). This survey indicates that the *A. niger* var. *niger* was the major species isolated from these two plants, with *A. foetidus* and *A. japonicus* occurring as minor species. Peanuts accounted for three species, while only two were isolated from maize. The work is similar to that of Magnoli *et al.* [16] who found that of the black aspergilli, *A. niger* var. *niger* along with *A. japonicus* var. *japonicus* was isolated from surface disinfected maize kernels from Argentina.

## 3. Mycotoxins Produced by Black *Aspergillus* sp.

The earliest report of toxicity from the black *Aspergillus* species was by Frischbier and Richtesteiger [17] who reported on the experimental poisoning of pigs fed bread that was inoculated with *A. niger* and that the toxic component was oxalic acid. Later, Wilson and Wilson [18] indirectly indicated *A. niger* as the toxic organisms since oxalic acid was isolated from moldy feedstuffs that was toxic to livestock. Presently, the type of the section, *A. niger*, has important industrial application and most strains of this specific species hold the Generally Recognized as Safe status issued by FDA. What is not clear, however, what species is intended when the term “A. niger” is used for all black spored aspergilli. Thus, correct taxonomic descriptions of species within this group are extremely important as this can serve to distinguish those that are phytopathogens and mycotoxic from those that have technological applications.

### 3.1. Ochratoxins

Ochratoxin A is the more toxic of two dihydroisocoumarins initially isolated from broth cultures of *A. ochraceus*. The other is ochratoxin B, which is the dechlorinated analog of ochratoxin A. Both A and B occur in smaller amounts as the methyl and ethyl esters. Ochratoxin A is one of the world’s most important mycotoxin, rated third of the top six, primarily due to the documented deaths of humans, primarily in Europe. The International Agency for Research on Cancer classifies this mycotoxin in the 2B group, possible carcinogen to humans [19], but it is not regulated in the United States, although it is in Europe (FAO Food and Nutrition Paper No. 81, 2004). Ochratoxin A is nephrotoxic, teratogenic, carcinogenic, and immunosuppressive in animals, and it is cytotoxic in hepatic cell lines. In humans, ochratoxin A has been associated with the Balkan Endemic Nephropathy, a tubule- interstitial nephropathy leading to a chronic renal failure that is characterized by high concentration of ochratoxin A in blood serum and urine of patients suffering from this disease [20]. 

In addition to the above *in vivo* description of toxicity from ochratoxin A, there is a recent finding of its mode of action at the cellular level. This toxin is reported to interact with tight junction pores, which regulate paracellular transport across cell and tissue membranes, by altering the four isoforms of cell-to-cell specific cell membrane adhesion proteins called claudins [21]. Thus, this toxin affects cell membrane integrity, producing non-regulated transports in and out of cells. This can have a high economic impact at the agricultural level effecting food products ranging from eggshell damage to increase bacterial infections. 

**Table 2:** Field collection of isolates with predicted species results depicted in Figure 1 [15].

Strain Number^a^	Location	Species
RRC 453	Peanut, South Georgia	*A. niger*
RRC 454	Peanut, South Georgia	*A. foetidus*
RRC 455	Peanut, South Georgia	*A. niger*
RRC 456	Peanut, South Georgia	*A. niger*
RRC 457	Peanut, South Georgia	*A. niger*
RRC 458	Peanut, South Georgia	*A. foetidus*
RRC 459	Peanut, South Georgia	*A. niger*
RRC 460	Peanut, South Georgia	*A. japonicus*
RRC 475	Peanut slurries, Dawson, Georgia	*A. niger*
RRC 476	Peanut slurries, Dawson, Georgia	*A. niger*
RRC 477	Peanut slurries, Dawson, Georgia	*A. foetidus*
RRC 478	Peanut slurries, Dawson, Georgia	*A. niger*
RRC 479	Peanut slurries, Dawson, Georgia	*A. japonicus*
RRC 480	Peanut slurries, Dawson, Georgia	*A. niger*
RRC 481	Peanut slurries, Dawson, Georgia	*A. niger*
RRC 482	Maize kernels, Midwestern, USA	*A. niger*
RRC 483	Maize kernels, Midwestern, USA	*A. niger*
RRC 484	Maize kernels, Midwestern, USA	*A. niger*
RRC 485	Maize kernels, Midwestern, USA	*A. niger*
RRC 486	Maize, kernels, Midwestern, USA	*A. niger*
RRC 487	Maize kernels, Midwestern, USA	*A. niger*
RRC 488	Maize kernels, Midwestern, USA	*A. niger*
RRC 489	Maize kernels, Midwestern, USA	*A. niger*
RRC 490	Maize kernels, Midwestern, USA	*A. niger*
RRC 493	Maize kernels, Midwestern, USA	*A. niger*
RRC 494	Maize kernels, Midwestern, USA	*A. niger*
RRC 495	Maize kernels, Midwestern, USA	*A. niger*
RRC 497	Maize kernels, Midwestern, USA	*A. niger*
RRC 500	Maize kernels, Midwestern, USA	*A. niger*
RRC 501	Maize kernels, Midwestern, USA	*A. niger*
RRC 503	Maize kernels, Midwestern, USA	*A. niger*
RRC 504	Maize kernels, Midwestern, USA	*A. niger*
RRC 507	Maize kernels, Midwestern, USA	*A. foetidus*
RRC 510	Maize kernels, Midwestern, USA	*A. niger*

^a^RRC, Russell Research Center culture collection, Athen, Ga.

### 3.2. Ochratoxin contaminated products and producing species

Ochratoxin A was isolated as a natural contaminant from maize [22], and since this report it has been isolated from many agronomically important crops, and processed food and feed. Commodities contaminated with this toxin predominantly include cereal and derived products including maize, wheat, rice, sorghum, mixed livestock, and poultry feed. Ochratoxin has also been found in green coffee beans, peanuts, olives, beer, grapes, raisins, peas, beans, barley, oats, rice, and wheat. It has been detected in processed food such as cheese, wine, grape juice, powdered milk, fruits, wine black pepper, and grape juice [8,23,24,25]. Ochratoxin A is isolated from meats of poultry and swine consuming contaminated feed [26], and has been detected in human blood and milk [27,28,29]. This indicates the widespread occurrence, and saprophytic and or parasitic nature of the black *Aspergillus* species. 

*Aspergillus niger* var. *niger* and *A. carbonarius* are two major producers of ochratoxin A. For example, 25% to 100% of the isolates of *A. carbonarius* are ochratoxigenic, and 0.6 to 50% of the isolates of *A. niger* var. *niger* are ochratoxigenic [13]. Recent surveys indicate that these two black-spored aspergilli are the main source of ochratoxin A in major food products, including maize and wheat, in both tropical and subtropical zones of the world [30,31]. Curiously, the *A. niger* strains that do produce ochratoxin have been placed in the type N RFLP pattern, while ochratoxin production is not associated with strains from the type T RFLP stains [32]. However, this difference might reflect nutritional requirement in culture as to those required on natural substrates [33]. Both *A. niger*, var. *niger* and *A. carbonarius* are common in the United States [9,15], along with other ochratoxigenic species, *A. foetidus*, and *A. tubingensis*, but their ability to produce the ochratoxins is unknown. However, the species as currently defined that occur on maize and peanuts or grapes have not been identified, nor has ochratoxin production been established on maize or peanuts by these species.

In the past, the occurrence of ochratoxin A in maize has always indicated that the maize was contaminated during storage and that the maize was grown in a temperate climate because the fungi that produce it, mainly *A. ochraceus*, *Penicillium verrucosum*, and *P. nordicum*, [34,35], grow well under cool to cold conditions. Currently, additional temperate species are indicated as being able to produce ochratoxin A and B. These include *A. alliaceus*, *a. sclerotiorum*, *A. sulphureus*, *a. albertensis*, *A. auricomus*, *A. melleus*, *A. glaucus*, *A. wentii*, *Neopetromyces muricatus*, and *A. westerdijkiae* [34,35,36] Members of a different *Aspergillus* section, the *Aspergillus* section *Nigri* (formerly known as *A. niger* aggregate) have now been isolated from maize and peanuts (Table 2). The *Aspergillus* section *Nigri* group occurs in the warmer temperate and tropical zones [9]. The highest percentages of ochratoxigenic strains were found within the *A.* section *Nigri* [37], the taxa responsible for the main source of ochratoxin A in animal feeds, especially in locations with less than desirable storage and humidity-temperature conditions [20,38]. Surveys of poultry feed and the poultry environment indicated that the most frequently isolated fungus, second to *F. verticillioides*, was a black-spored species of the *A. niger* aggregate [39,40]. Maize and mixed diets prepared with maize, especially for poultry, contain a high number of *A. niger* CFU [39,40,41]. In Georgia, a black spored species, e.g., *A. niger* var. *niger*, is a major pathogen of peanuts, causing seedling blight of all the major cultivars. However, there are additional species that are found in lesser frequencies (Table 3) [15]. The problem is compounded because maize is a rotational crop for peanuts in several areas of the state, which results in infected maize. Neither the precise identify of the pathogen nor has the biological nature of this association been defined. Thus, ochratoxin A potentially can occur throughout the temperate, tropical, and subtropical climates of the world. 

### 3.3. Fumonisins

Additional toxic substances are presented in Table 1. It was recently discovered that four strains of *A. niger* were able to produce another type of mycotoxins, the fumonisins, which are commonly associated with strains of the maize pathogen *Fusarium verticillioides (Gibberella moniliformis)* and other *Fusarium* species [42]. *Fusarium verticillioides*-contaminated maize is correlated with human esophageal cancer, and the fumonisins are highly toxic to horses, pigs, and poultry [43]. The fumonisin mycotoxins are carcinogenic, although B_2 _is more cytotoxic than B_1_. The most commonly isolated fumonisin is the B_1 _homologue, while B_2 _is isolated less frequently. Only strains of *A. niger* var. *niger* have currently been shown to produce the fumonisins including B_4_ and the new B_6_ homologue [34,37,44]. So far only the strains of *A. niger* var. *niger* from grapes have been reported as producers of the fumonisins. A survey of black aspergilli isolated from raisins indicated that 77% of *A. niger* from grapes produced fumonisin B_2 _and B_4_, and interestingly none of these strains produced the ochratoxins [34,44], suggesting that the ability to produce each mycotoxin is entirely separate of conditions and depends on the genetics of a strain. Recently, another fumonisin homologue, fumonisin B_6_, was isolated from *A. niger*, with in general the frequencies of production of fumonisins in this species so far are 100% FB_2_, 10–25% FB_4_, and 5–10% FB_6_[37]. The black aspergilli are co-isolated with *F. verticillioides* from maize and peanuts suggesting that the source of fumonisin accumulation on these substrates might be derived from either fungus. However, the production of strains of *A. niger* var. *niger* from maize has not been demonstrated. Since the black aspergilli, especially *A. niger* var. *niger*, are used in so many biotechnological processes for food use, the production of the fumonisins by the commonly occurrence of this species increases the concern for food safety.

**Table 3:** Species of black spored aspergilli isolated from surface sterilized maize kernels and peanuts samples obtained from the Midwest, and South Georgia, USA.

Aspergillus Species	% Isolation Frequency^a^	
	Maize	Peanuts
*A. niger*	95	67
*A. foetidus*	-	20
*A. japonicus*	5	13

^a ^Data modified from Palencia *et al.* [15].

### 3.4. Other mycotoxins

The black aspergilli have produced a variety of biologically active compounds [36], some of which are phytotoxic. One not indicated as being produced by the black aspergilli in the extensive review is penicillic acid, which we consider important not only for it biological activity as a mycotoxin but also due to its numerous other properties. These properties are reviewed with the hope that attention is drawn to it as a metabolite of the black aspergilli. Penicillic acid (γ -keto- β-methoxy- δ-methylene-A^α^ –henenoic acid) is particularly produced by most of the golden colored species of section *Circumdati* (=*A. ochraceus* group), and is oftentimes co-produced with ochratoxin A by strains of *A. ochraceus* [40,45].Besides the *A. ochraceus* group, penicillic acid is also produced by numerous other species of *Aspergillus* and *Penicillium* [45,46,47], which suggest that penicillic acid is ubiquitous in strains and species of other subsections of these two genera. Penicillic acid is mycotoxic and synergistic with ochratoxin A in several animal studies [48,49,50], and phytotoxic to seedlings [51,52]. While the production of penicillic acid has not been reported in the most recent review of secondary metabolites reported in the black aspergilli [36], its production might explain a role for this species in this subsection as pathogens in several seedling diseases. 

Penicillic acid is also inhibitory with microorganisms, particularly Gram-negative bacteria [53], and may be intricately involved with competition and ecological success of producing species. Recently, penicillic acid was established as an effective quorum sensing inhibitor [54,55], therefore interfering with cellular communication, and producing disruptive effects on virulence expression by pathogenic species, especially Gram-negative species. Its mode of action with bacteria might reflect it activity as an inhibitor of quorum sensing. Quorum sensing affects cellular activities of bacteria by interfering with several aspects of bacterial metabolism ranging from those necessary for growth to those responsible for cellular motility [54,56] to biofilm formation [57]. However, it is argued that quorum sensing is highly specific and it probably cannot be broadly applied to control all bacteria where quorum sensing is expressed [58]. This range of biological activity for penicillic acid indicates several desirable pharmacological benefits such as quorum sensing to undesirable one such as an inhibitor to bacteria used as biocontrol agents against penicillic acid positive pathogenic fungi. Penicillic acid therefore should be viewed also as an antibiotic where it mode of microbial antagonism is one of interfering with important signaling mechanisms used by colonizing bacteria, conceivably reducing their competition with the producing *Aspergillus* species. Since the biological activities described for penicillic acid above are important from several control measures, its production by the black aspergilli should be investigated.

## 4. Host Associations and Plant Pathology

Black aspergilli are reported as pre- and post-harvest pathogens in maize, other cereal grain, bunch grapes, onions, garlic, soya beans, apples, mangoes, and peanuts [16,41,59,60,61], although the extent of damage on each host depends on unknown predisposing environmental factors. The inoculum source of most species is the soil and litter, particular the vineyards soils [9,62,63,64]. Other sources include the seed of most crops [15,62] or fruit [63]. Most black aspergilli are indicated as opportunistic pathogens of fruits such as grapes and some spices [62,63]. In a small survey [15], several species of black aspergilli were isolated from surface disinfected maize and peanuts, which indicated that these species were endophytes. Endophytic associations have also been reported in onions and garlic [62], although these were characterized as latent infections. The better term for these infections is symptomless, as endophytic infections have proved in all instances as not latent or dormant as implied, but metabolically active, colonizing the host producing several classes of secondary metabolites, some of which are toxic. The major difference is the absence of disease symptoms produced during these infections. These endophytic infections can become, however, pathogenic under some biotic and abiotic conditions either pre- or post-harvest. Symptomless infections pose grave problems from a food safety concern, as commodities contaminated by such infections are not obvious, appear normal, but can contain toxic metabolites.

**Figure 2:**
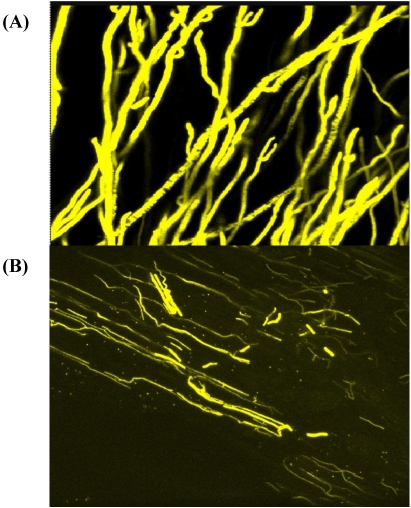
*Aspergillus carbonarius* SRRC2131 transformed with yellow fluorescent protein growing on potato dextrose agar medium (A) and in the roots of maize seedlings, illustrating the symptomless endophytic colonization of maize following the soil inoculation with the fungus (B), which persists even under drought conditions [65].

An investigation of the endophytic nature of maize seedling was investigated. *Aspergillus carbonarius SRRC 2131 and A. niger* var. *niger* SRRC 13 were transformed with a yellow fluorescent protein vector that was used to measure colonization of maize seedlings [15,65]. The transformed black aspergilli did not affect the ability of these strains to colonize maize seedling (Figure 2). These transformed species were isolated from surface disinfected seedling roots and leaves of plants grown under ideal growth room conditions, and there were no significant differences in seedling height and stem thickness [15]. The infection remained symptomless and attempts at inducing seedling blight by inducing drought did not produce the disease. The endophytic nature of the black aspergilli was indicated from the recovery of *A. niger* from surface disinfected plant materials of onions by Hayden and Maude [62]. The infection in onion was also symptomless and infection was proved to develop from contaminated onion seed. Seedling onions were, as in the maize seedlings, similar to non-infected except in shoot length [62]. 

**Figure 3:**
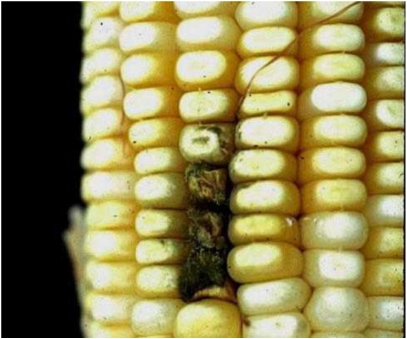
Maize kernel rot produced by *Aspergillus niger.*

**Figure 4:**
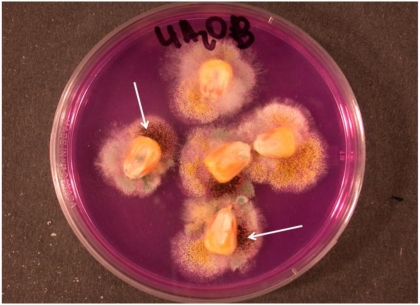
Isolation of black *Aspergillus sp* from surface disinfected field maize kernels, showing a black *Aspergillus* species (arrows) along with *A. ochraceus* growing from maize kernels on an isolation medium.

In grapes, another situation is described. *Aspergillus* rot in grapes is caused by the black species, *A. niger*, var. *niger*, *A. carbonarius* and *A. aculeatus* [63]. These black aspergilli are reported as opportunistic pathogens of damaged berries of grapes, since in the absence of damaged spores remain over the surface of grapes without causing visible pathology. Further, *A. niger* is known to cause kernel rot of maize (Figure 3), which is similar to ear/kernel rot produced by *Fusarium verticillioides*, and *F. graminearum*. However, black aspergilli are isolated from surface disinfected kernels of maize (Figure 4). Symptomless infections in onion by *A. niger* var. *niger* can develop into a postharvest disease, but perhaps due to injury to the bulbs or unsuited storage conditions [62,66]. In the case of maize, infection and the resulting kernel rot may occur from wounds produced by earworms and other damaging insects, similar to that which occurs from infections by *A. flavus.* Nevertheless, endophytic infections do occur as demonstrated from isolations from surface disinfected kernels, and these endophytic infections can colonize plant tissue. Therefore, some black aspergilli are capable of a biotrophic endophytic existence with maize and onion. Since these two plants are widely separated taxonomically, it is possible that endophytic infection by the black aspergilli exist in numerous plant taxa.

Since ochratoxin A is present in maize, and other cereal grain under field conditions [16], there is a suggestion that the black aspergilli species may form some relationship with maize during its growth under field conditions or that it is interactive with other field fungi. In a laboratory study, it was demonstrated that the fumonisin-producing fungi, *F. verticillioides*, can exclude the *Aspergillus* species such as *A. niger var. niger*, from colonizing maize, but this depended on optimum temperature and specific water activity levels [67,68]. The interaction of antagonistic substances was not examined in that study, but conceivable competition may also relate to inhibitory substances as well as the abiotic factors measured. 

## 5. Conclusions

In the past most descriptions and concerns relative to the black aspergilli have been referred to as *A. niger*, resulting in a great deal of confusions concerning the actual species observed. Recent molecular analysis of the black aspergilli indicated several cryptic species, which now should be aligned with recent phytopathological and toxicological concerns. To prevent this confusion, the older *A. niger* has been replaced by *A. niger*, *var. niger* in order to exclude the prior confusion in discussions of both the earlier and recent discriptions. The subgenus *Circumdati* section *Nigri* consists of at least 19 species of black spore species of which *A. niger* var. *niger* is probably the most dominant species in the US where it is found in most soil types and on dozens of fruits and cereal grains. Of major concern is the relationship of the black aspergilli with maize and peanuts, two plants of major economic concern in the US Strains of *A. niger* var. *niger*, along with others, can produce the ochratoxins and the fumonisin B_1_ mycotoxins. In addition to the effect of the mycotoxins on animals and humans, these toxins are interactive with other secondary metabolites to produce synergistic effects. Others are also phytotoxic [69], suggesting role for this mycotoxin in the pathology of seedling blight of peanuts and maize. The production of both the ochratoxins and the fumonisins by the black aspergilli extends the concern since food and feeds not inductive to one mycotoxin can be inductive to the other. Similarly, attempts at control of one might not control the other. Current disease descriptions are based on the casual identification of the black aspergilli as belonging to the older descriptions of *A. niger.* Certain species have been characterized as producing specific toxins and associated pathology. This indicates the need for reexamination of taxa associated with specific food crops. Additionally, detailed studies of their host relationship are also indicated, as some species are associated with crops as symptomless endophytes. There are also reports on the co-occurrence of both ochratoxin A with the fumonisins in maize [70,71], which suggests interactions between producing endophytic fungi on maize, such as the maize endophyte *F. verticillioides*. Further, evidence of co-infection of *A. niger* with *A. ochraceus* in maize is observed also a concern as this presents difficulty in identifying the correct offending mycotoxic species. The black aspergilli are known pathogens in peanut culture where major problems are indicated from the seedling stage or during peanut set late in matured plants. The result is poor quality nuts that are rated too low for human consumption. Thus, pathogenic expressions in maize, cotton, grapes, peanuts and other plants, the production of the ochratoxins and fumonisins on these products, and the unknown species associated with specific pathological expressions on these important crop plants underlines the need for further studies of the black aspergilli. Finally, the prediction of rising global temperatures should influence the population patterns and shifts of species within the *A. niger* group to more northern latitudes that should increase the number of additional crops, adding to the mycotoxigenic potential globally for all species of the subgenera *Circumdata.*
